# Generation of high-resolution MPRAGE-like images from 3D head MRI localizer (AutoAlign Head) images using a deep learning-based model

**DOI:** 10.1007/s11604-024-01728-8

**Published:** 2025-01-11

**Authors:** Hiroshi Tagawa, Yasutaka Fushimi, Koji Fujimoto, Satoshi Nakajima, Sachi Okuchi, Akihiko Sakata, Sayo Otani, Krishna Pandu Wicaksono, Yang Wang, Satoshi Ikeda, Shuichi Ito, Masaki Umehana, Akihiro Shimotake, Akira Kuzuya, Yuji Nakamoto

**Affiliations:** 1 Department of Diagnostic Imaging and Nuclear Medicine, Graduate School of Medicine, Kyoto University, 54 Shogoin Kawahara-Cho, Sakyo-Ku, Kyoto, 606-8507 Japan; 2 Department of Advanced Imaging in Medical Magnetic Resonance, Graduate School of Medicine, Kyoto University, Kyoto, 606-8507 Japan; 3 Department of Radiology, University of Indonesia, Jakarta Pusat, Indonesia; 4 Department of Neurology, Graduate School of Medicine, Kyoto University, Kyoto, 606-8507 Japan

**Keywords:** Magnetization prepared rapid gradient echo, Voxel-based morphometric analysis, Generative adversarial network, Machine learning

## Abstract

**Purpose:**

Magnetization prepared rapid gradient echo (MPRAGE) is a useful three-dimensional (3D) T1-weighted sequence, but is not a priority in routine brain examinations. We hypothesized that converting 3D MRI localizer (AutoAlign Head) images to MPRAGE-like images with deep learning (DL) would be beneficial for diagnosing and researching dementia and neurodegenerative diseases. We aimed to establish and evaluate a DL-based model for generating MPRAGE-like images from MRI localizers.

**Materials and methods:**

Brain MRI examinations including MPRAGE taken at a single institution for investigation of mild cognitive impairment, dementia and epilepsy between January 2020 and December 2022 were included retrospectively. Images taken in 2020 or 2021 were assigned to training and validation datasets, and images from 2022 were used for the test dataset. Using the training and validation set, we determined one model using visual evaluation by radiologists with reference to image quality metrics of peak signal-to-noise ratio (PSNR), structural similarity index measure (SSIM), and Learned Perceptual Image Patch Similarity (LPIPS). The test dataset was evaluated by visual assessment and quality metrics. Voxel-based morphometric analysis was also performed, and we evaluated Dice score and volume differences between generated and original images of major structures were calculated as absolute symmetrized percent change.

**Results:**

Training, validation, and test datasets comprised 340 patients (mean age, 56.1 ± 24.4 years; 195 women), 36 patients (67.3 ± 18.3 years, 20 women), and 193 patients (59.5 ± 24.4 years; 111 women), respectively. The test dataset showed: PSNR, 35.4 ± 4.91; SSIM, 0.871 ± 0.058; and LPIPS 0.045 ± 0.017. No overfitting was observed. Dice scores for the segmentation of main structures ranged from 0.788 (left amygdala) to 0.926 (left ventricle). Quadratic weighted Cohen kappa values of visual score for medial temporal lobe between original and generated images were 0.80–0.88.

**Conclusion:**

Images generated using our DL-based model can be used for post-processing and visual evaluation of medial temporal lobe atrophy.

**Supplementary Information:**

The online version contains supplementary material available at 10.1007/s11604-024-01728-8.

## Introduction

Magnetization prepared rapid gradient echo (MPRAGE) is a high-resolution three-dimensional (3D) T1-weighted imaging with inversion recovery (IR) pre-pulse [1], offering excellent T1-weighted contrast and good visualization of the cortico-medullary boundaries of the brain. With MPRAGE, differences in image quality between different machines are relatively small [2]. For this reason, MPRAGE is widely used for research into brain morphology analysis and the diagnosis of dementia and neurodegenerative diseases [3–6]. However, T1-weighted images, including MPRAGE, are not a priority in many examinations, such as evaluations for cerebrovascular disorders [7, 8]. Data acquisition is therefore often omitted due to practical limitations on examination time. In clinical practice, where contrast-enhanced T1-weighted imaging is performed, 3D turbo spin echo sequences and 3D gradient echo sequences are preferred instead of MPRAGE for the better contrast enhancement or shorter acquisition time [7–9]. Retrospective analysis of brain morphology with MPRAGE imaging is therefore often difficult.

Advances in deep learning (DL) have enabled the generation of various images or the conversion of images from one domain to another using the idea of the generative adversarial network (GAN) [10–12]. DL is also now being used for image transformation of medical images [13]. Studies have examined transformation between different MRI contrasts or between different imaging modalities [14–17]. For analyses of brain morphology, a DL-based method has also been reported using MR images taken in clinical practice, such as two-dimensional FLAIR, instead of high-resolution T1-weighted images [18]. Although the generation of similar images using DL-based image transformation has become technically feasible, radiological validation of the generated images is not yet sufficient, representing a significant barrier to clinical application. In DL-based image conversion, the relationship between two domains is captured by the generator network. While necessary information may be missing from the source images or model, the network makes up for such information in the process of image conversion without consideration of radiological correctness. In other words, DL-based image transformations, such as inter-modality transformations, rely heavily on trained networks. These processes are thus considered to carry a high risk of false conversion and to require careful evaluation.

The MRI localizer has T1-weighted contrast and is always taken in each MRI examination to determine slice positioning. AutoAlign Head (AAH) is a 3D FLASH sequence for Siemens' head MRI localizer, enabling automatic slice positioning in a very short acquisition time of less than 15 s [19]. We assumed that converting AAH to MPRAGE with DL would have less trouble compared to inter-modality transformation because of the relatively similar image content. The ability to create MPRAGE images from localizer images would enable research and clinical use based on brain morphometric analyses, which until now have been difficult for cases without MPRAGE scans.

This study aimed to establish a DL-based model for generating MPRAGE images from MRI localizers. To ensure sufficient quality for clinical and research use, we evaluated the usefulness and validity of the model by software-based voxel-based morphometry (VBM) and visual evaluation by radiologists.

## Materials and methods

### Subjects and datasets

This study used brain MR images for screening or workup of mild cognitive impairment (MCI)/dementia and epilepsy, including MPRAGE, taken at a single institution between January 2020 and December 2022. In this study, patient selection criteria for MCI/dementia meet the clinical diagnostic criteria for probable Alzheimer's disease (AD) dementia and MCI due to AD based on the 2011 guidelines of the National Institute on Aging and the Alzheimer's Association. Clinical diagnosis was performed by board-certified neurologists. Regarding epilepsy, patients with epilepsy diagnosed by board-certified neurologists underwent an “epilepsy MRI protocol” including MPRAGE [20]. In the other MRI protocols, MPRAGE was not used at our institution due to MRI examination slots. We excluded cases showing excessive motion artifacts or those in which the default resolution of MPRAGE had been changed to adjust skull size. For the test dataset, patients included in the training and validation datasets were excluded to avoid affecting evaluations of the test dataset. Images taken between 2020 and 2021 were assigned to training and validation datasets at a ratio of 9:1. All images from 2022 were used for the test dataset because it was difficult to use datasets from other institutes or public datasets due to the use of localizer images and the need to increase the number of cases to verify clinical utility. This retrospective study was approved by the institutional ethics committee. All MR images had only been taken out of clinical necessity, and the need to obtain written informed consent was waived based on the retrospective nature of the work. No subjects overlapped with previously published work.

### MRI acquisition

Acquisitions were performed using 3.0-T whole-body systems (Magnetom Skyra, Prisma, and Vida; Siemens Healthineers, Erlangen, Germany) using 32-, 64-, and 32-channel receive-only head coils, respectively. Imaging parameters were as follows. AAH (3D FLASH): TR, 3.15 ms; TE, 1.37 ms; flip angle (FA), 8°; bandwidth, 540 Hz/pixel; spatial resolution, isotropic voxels of 1.6 mm; and slices, 128. For acceleration, 24 reference lines were acquired in the phase-encoding direction, and generalized autocalibrating partially parallel acquisition (GRAPPA) 3 × was used. The acquisition time was 14 s. MPRAGE: TR, 2300 ms; TE, 4.67 ms; FA, 9°; bandwidth, 130 Hz/pixel; spatial resolution, isotropic voxels of 0.9 mm, and slices, 208. For acceleration, 24 reference lines were acquired in the phase-encoding direction, and GRAPPA 2 × was used. The acquisition time was 4 min 26 s.

### Deep learning model and training

We used the pix2pix method, which combines a U-Net generator with a conditional GAN [10]. Pix2pix is widely used for image-to-image translation and has reportedly been used for medical images [18, 21, 22]. We modified the pix2pix code in pytorch (https://github.com/junyanz/pytorch-CycleGAN-and-pix2pix) [10]. Details of the model modification and training procedure are described in the Supplementary material. The code used in this study is available on GitHub (https://github.com/kuponuga/aah2mprage).

### Image evaluation metrics

In training, validation, and testing, we used peak signal-to-noise ratio (PSNR, with higher values considered better), structural similarity index measure (SSIM, with higher values considered better) [23], and Learned Perceptual Image Patch Similarity (LPIPS, with lower values considered better) [24] (https://github.com/richzhang/PerceptualSimilarity) as metrics for assessing image quality. The default Alex network was used in LPIPS.

### Model selection

Two radiologists (A.S. and H.T., with 15 and 10 years of experience in neuroradiology, respectively) performed visual evaluation of generated images and determined the best model. First, we extracted eight slices that included the brain, reviewed the images for each condition, and excluded those that showed inaccurate translations or apparent artifacts. We then chose those with superior image quality based on visual evaluation and finally selected the best model based on a consensus of two radiologists. For the selected model, we looked through all the validation images to check for any abnormalities that would cause significant problems for evaluation. Image evaluation indices (PSNR, SSIM, LPIPS) were also calculated to see if they differed from the radiologists’ evaluations.

### Tests

#### Objective image evaluation

PSNR, SSIM, and LPIPS were calculated between the original MPRAGE and generated images from the test datasets only for those sections containing brain parenchyma. As with the training procedure (Supplementary material), one radiologist (H.T.) excluded those slices not containing brain parenchyma.

#### VBM

VBM analysis was performed using FreeSurfer (version 7.4.1, https://surfer.nmr.mgh.harvard.edu/fswiki/FreeSurferWiki). First, image processing and segmentation were performed on the original MPRAGE and on generated images using the FreeSurfer “recon-all” command. Considering the influence of manual correction on analysis results, we excluded images from evaluation without manual correction if an error was identified. Concordance of the segmentation regions of major structures (each side of the thalamus, caudate, putamen, hippocampus, amygdala, cerebral white matter, cerebral cortex, and lateral ventricle, third ventricle, and fourth ventricle) was evaluated using Dice scores. These Dice scores were calculated using the “mri_overlap” command on FreeSurfer. We also evaluated volume differences as the absolute symmetrized percent change (ASPC). We used the volumes provided from FreeSurfer recon-all stats. Dice score and ASPC are defined as follows:

$$Dice\left(X, Y\right)= \frac{2\left|X \cap Y\right|}{\left|X\right| + \left|Y\right|}$$

$$ASPC\left(X, Y\right)=\frac{200\left|X-Y\right|}{\left|X\right| + \left|Y\right|}$$

#### Visual evaluation

Three radiologists (S.Ik., S.It., and M.U., each with 7 years of experience in neuroradiology) evaluated both MPRAGE and generated images of the test dataset obtained in 2022. The presence of medial temporal lobe atrophy (MTA) and old cerebral infarction or hemorrhage was visually assessed. The following medial temporal lobe atrophy score [25] was used to evaluate atrophy: 0, normal; 1, widened choroid fissure; 2, increased widening of the choroid fissure, widening of the temporal horn, opening of other sulci (i.e., collateral/fusiform sulcus); 3, pronounced loss of hippocampal volume; and 4, end-stage atrophy. Cerebrovascular lesions were defined as those with a short diameter ≥ 1 cm. Generated images were evaluated first. Four weeks later, MPRAGE images were evaluated next. Raters were not informed which images were the generated images and which were MPRAGE, and the order of cases was randomized. For lesions not detected on generated images, another radiologist (H.T.) reviewed the images. Conspicuous artifacts were also recorded. For MTA scores, weighted Cohen kappa was calculated between MPRAGE and the generated images. Quadratic weighting was used to emphasize the large difference in scores. Statistical analysis was performed with R (version 4.3.1, https://www.r-project.org/) on RStudio (version 2023.09.1, https://posit.co/download/rstudio-desktop/).

## Results

### Characteristics of the study population

**Fig. 1:**
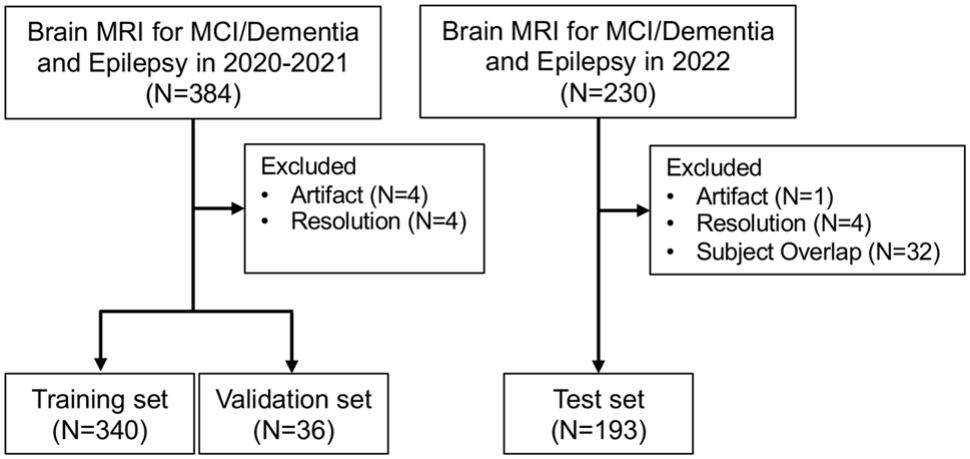
Inclusion and exclusion flowcharts for datasets. MCI, mild cognitive impairment

A total of 384 eligible MRI examinations were performed in 2020 and 2021, and 230 were performed in 2022. Eight examinations were excluded from the training and validation datasets due to artifacts (n = 4) and different resolution (n = 4), and a total of 376 examinations were finally included (training, n = 340; validation, n = 36). Thirty-seven examinations were excluded from the test datasets due to artifacts (n = 1), different resolution (n = 4), and subject overlap (n = 32). A total of 193 examinations were included for the test dataset (Fig. 1). Details of the patients included in each dataset are provided in Table 1.

**Table 1:** Characteristics of patients and datasets

Datasets		Train	Validation	Test
Patients (female)		340 (195)	36 (20)	193 (111)
Mean age ± SD (range)		56.1 ± 24.4 (2–95)	67.3 ± 18.3 (15–93)	59.5 ± 24.4 (4–92)
No. of image pairs (including brain)		87,040 (47,880)	9216 (5259)	49,408 (28,460)
Indication for study				
Epilepsy		178	15	88
MCI/Dementia		162	21	105
Clinical diagnosis	AD	46	7	23
	VaD	9	4	8
	AD + VaD	3	0	0
	DLB	8	0	9
	FTLD	3	0	2
	iNPH	3	0	1
	Others	2	1	0
	Not specified	88	9	63

MCI, mild cognitive impairment; AD, Alzheimer’s disease; VaD, vascular dementia; DLB, dementia with Lewy bodies; FTLD, frontotemporal lobar degeneration; iNPH, idiopathic normal-pressure hydrocephalus. Note that “No. of image pairs” represents the total number of slice pairs of MPRAGE and AAH. Only images that included the brain were used for training and evaluated by image metrics for validation and test datasets

### Training and validation

**Fig. 2:**
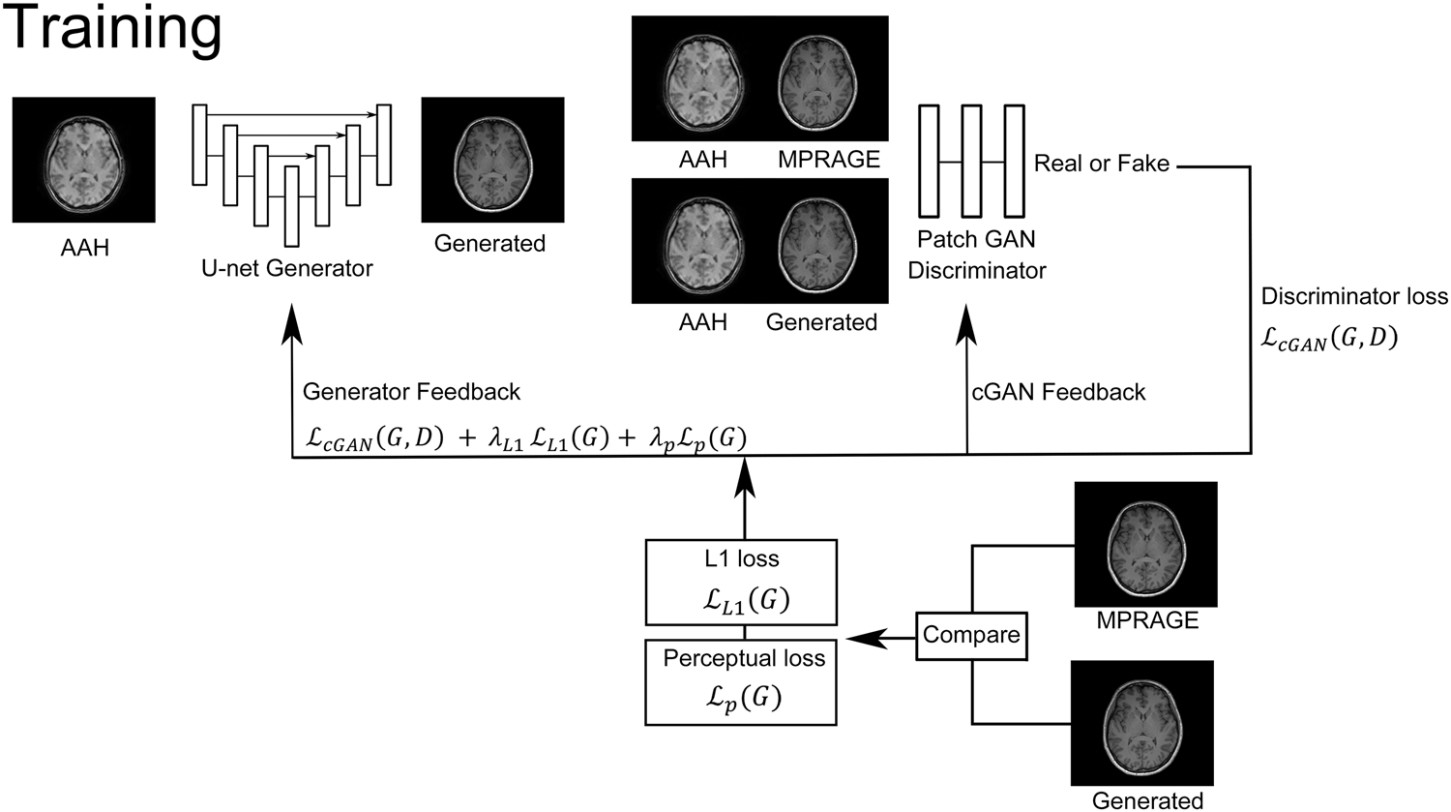
Deep learning procedures. AAH, AutoAlign Head; cGAN, conditional generative adversarial network

DL procedures are presented in Fig. 2. Thirty different models were created by setting ${\lambda }_{L1}$ to 100, 699, 1000, 6999, and 10,000, and ${\lambda }_{P}$ to 0, 100, 699, 1000, 6999, and 10,000, where ${\lambda }_{L1}$ represents L1 loss weights and ${\lambda }_{P}$ represents VGG perceptual loss weights for the evaluation function (Supplementary material). The maximum number of epochs was set to 40, empirically determined based on the learning curve (Supplementary Fig. 1). We generated MPRAGE-like images from the validation dataset at 10, 20, 30, and 40 epochs for each model. The two radiologists visually evaluated the images and decided on the optimal model (${\lambda }_{L1}$, 699; ${\lambda }_{P}$, 699; and epochs, 30) by consensus. Sample images used to select the optimal model are shown in Supplementary Fig. 2. The mean and standard deviation (SD) of image evaluation metrics for each model are listed in Supplementary Tables 1–3. Image metrics of the final model were as follows: PSNR, 35.1 ± 5.04; SSIM, 0.873 ± 0.059; and LPIPS, 0.044 ± 0.017. PSNR and SSIM were ranked 76th and 71st out of 120 (4 types of epochs with 30 different parameters), respectively, while LPIPS was ranked 12th, relatively close to the evaluation of the radiologists. For reference, we compared the original MPRAGE and original AAH, showing image metrics of PSNR 32.1 ± 5.34, SSIM 0.787 ± 0.059, and LPIPS 0.136 ± 0.032.

### Objective image evaluation using the test dataset

**Fig. 3:**
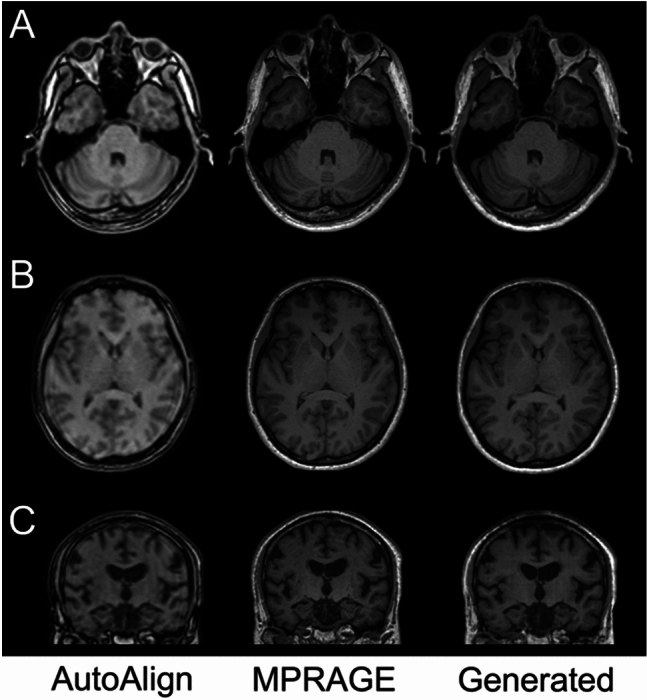
Representative images from the test datasets. Left: AutoAlign Head (AAH), head localizer image; middle: original MPRAGE; right: generated image. **A**, **B** Axial images. Training was performed on axial images. On AAH and MPRAGE, images were reconstructed from sagittal section images. **C** Coronal image for evaluation of medial temporal lobe atrophy. On AAH and MPRAGE, images were reconstructed from sagittal images. The generated image was created as axial images. Whole-brain images for A are shown in Supplementary Fig. 3

Image metrics (mean ± SD) between MPRAGE and the generated images were as follows: PSNR, 35.4 ± 4.91; SSIM, 0.871 ± 0.058; and LPIPS, 0.045 ± 0.017. Image metrics in the test dataset were almost equivalent to those in the validation dataset (PSNR, 35.1; SSIM, 0.873; LPIPS, 0.044), and no overfitting was observed. Representative images of the test datasets are shown in Fig. 3.

### VBM

On FreeSurfer, the Recon-all function ran successfully without errors in both MPRAGE and generated images, so all cases included in test datasets were included in VBM analysis. Mean Dice scores of major structures volume ranged from 0.788 (left amygdala) to 0.923 (right ventricle), with higher values considered better. Mean ASPCs ranged from 12.00 (left pallium) to 2.23 (left ventricle), with lower values considered better. All values for Dice scores and ASPC are shown in Table 2. Dice score and percentage volume differences of the hippocampus from VBM were 0.834 and 6.18 for the left, and 0.840 and 5.54 for the right, respectively.

**Table 2:** Dice score and ASPC on the VBM study

		Thalamus		Caudate		Putamen		Pallidum		Hippocampus	
		Left	Right	Left	Right	Left	Right	Left	Right	Left	Right
Dice Score	Mean (SD)	0.893 (0.042)	0.893 (0.031)	0.842 (0.061)	0.840 (0.056)	0.818 (0.074)	0.825 (0.089)	0.793 (0.086)	0.797 (0.090)	0.834 (0.067)	0.840 (0.068)
ASPC	Mean (SD)	6.49 (5.65)	6.69 (6.71)	7.72 (7.51)	8.88 (8.07)	10.02 (7.06)	8.65 (9.12)	12.00 (10.00)	11.77 (10.66)	6.18 (6.86)	5.54 (7.59)

		Amygdala		Cerebral white matter		Cerebral cortex		Lateral ventricle		Third ventricle	Fourth ventricle
		Left	Right	Left	Right	Left	Right	Left	Right		
Dice Score	Mean (SD)	0.788 (0.103)	0.834 (0.084)	0.875 (0.027)	0.872 (0.022)	0.808 (0.036)	0.803 (0.034)	0.926 (0.033)	0.923 (0.032)	0.879 (0.029)	0.841 (0.032)
ASPC	Mean (SD)	11.62 (12.47)	8.28 (7.72)	7.23 (5.99)	7.90 (5.06)	3.00 (2.32)	3.24 (2.54)	2.23 (1.84)	2.91 (2.87)	5.45 (4.72)	7.11 (5.56)

ASPC, absolute symmetrized percent change

### Visual evaluation

**Fig. 4:**
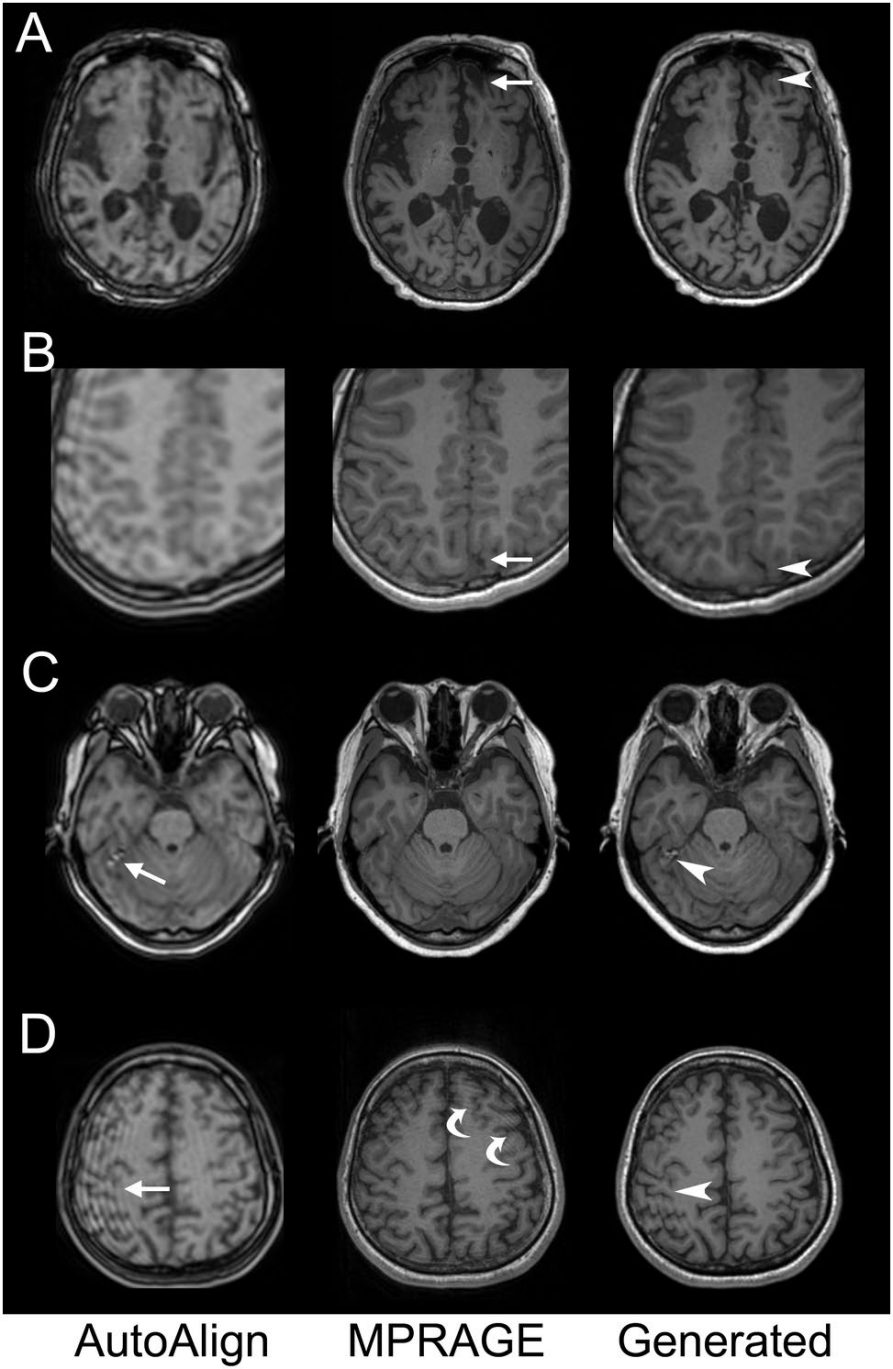
Representative images containing artifacts from test datasets. **A** MPRAGE shows an old infarction in the left frontal lobe (arrow), whereas the generated image shows normal-appearing cortex-like structures (arrowhead), making the infarction difficult to recognize. **B** On MPRAGE, the superior sagittal sinus (SSS) and neighboring brain parenchyma can be distinguished (arrow), but the SSS is converted to a structure mimicking brain parenchyma in the generated image (arrowhead). Note that the truncation artifact seen in the AutoAlign Head (AAH) head localizer image has been removed from generated images. **C** AAH shows a heterogeneous high-intensity artifact around the right tentorium (arrow); in the generated image, this artifact has not been removed (arrowhead). **D** AAH shows strong truncation artifacts (arrow); these artifacts remain in the generated image (arrowhead). Note that artifacts are also seen on MPRAGE due to motion artifacts (curved arrows)

Quadratic weighted Cohen kappa values for MTA scores between MPRAGE and generated images for each rater were 0.84 (95% confidence interval [CI] 0.81–0.87), 0.88 (95%CI 0.81–0.87), and 0.80 (95%CI 0.72–0.88). Intra-class correlation coefficients (ICC) (2,1) between raters were 0.731 (95%CI 0.599–0.813) for MPRAGE and 0.79 (95%CI 0.725–0.837) for the generated images. The breakdown of MTA scores and the number of detected cerebrovascular disorders are shown in Tables 3 and 4, respectively. A difference in the number of cerebrovascular disorders was detected between MPRAGE and generated images for all raters. A review of cerebrovascular lesions that could not be detected on generated images by visual evaluation revealed “normal-looking” translations, which complemented the lesion or structures other than the brain with the normal brain structure, in seven lesions (Fig. 4A, B). On the other hand, none of the lesions identified only in the generated images were clearly due to artifacts from the image conversion.

**Table 3:** Visual evaluation of medial temporal atrophy (visual scale score) by three radiologists

	Rater A		Rater B		Rater C	
Medial temporal atrophy (Visual scale score)	Original	Generated	Original	Generated	Original	Generated
0	116	145	180	203	202	176
1	98	98	99	98	99	123
2	96	91	72	56	51	47
3	47	28	22	18	13	12
4	29	24	13	11	21	28
Weighted Cohen κ (95%CI)	0.84 (0.81, 0.87)		0.88 (0.84, 0.91)		0.80 (0.72, 0.88)	

Rating protocol for the visual scale score of medial temporal atrophy: 0, normal; 1, widened choroid fissure; 2, increased widening of the choroid fissure, widening of the temporal horn, opening of other sulci (i.e., collateral/fusiform sulcus); 3, pronounced loss of hippocampal volume; 4, end-stage atrophy. Quadratic weighting was used for Cohen kappa

**Table 4:** Visual evaluation of cerebrovascular disease by three radiologists

	Rater A		Rater B		Rater C	
	MPRAGE	Generated	MPRAGE	Generated	MPRAGE	Generated
Detected lesions	27	22	32	15	30	28
Detected only on MPRAGE	11	–	17	–	9	–
Detected only on generated images	–	6	–	0	–	7

### Artifacts

In addition to “normal-looking” translations, several artifacts were noted in generated images (Fig. 4C, D). These were also observed in AAH and could not be eliminated by the model. On the other hand, truncation artifacts appeared to be adequately eliminated.

## Discussion

This study established a DL-based image-to-image translation model for generating MPRAGE-like images from head MRI localizer using image evaluation metrics and visual examination by radiologists. No apparent overfitting was observed in comparisons of image metrics for each dataset. We also demonstrated the reliability and validity of our model by VBM (FreeSurfer) and visual evaluation by radiologists. Dice scores for the segmentation of main structures on FreeSurfer ranged from 0.788 (left amygdala) to 0.926 (left ventricle) (Table 2). Quadratic weighted Cohen kappa values for the visual score of the medial temporal lobe between generated and original images were 0.80–0.88.

In FreeSurfer analysis, mean Dice score for segmentation results between MPRAGE and generated images was consistently above 0.8, with the exception of the pallidum and left amygdala. Agreement for segmentation between both types of image appeared to be excellent. Volume differences (ASPC) varied by structure, but were about 6% for the hippocampus. A longitudinal study revealed that patients with MCI who progressed to AD within 3 years showed hippocampal atrophy progressing by about 4% per year [26]. Our model might not have been sufficiently accurate to assess MCI patients, but was considered acceptable for use in the diagnosis of AD. Since volume differences for the putamen, pallidum, and amygdala were about 10%, more accurate modeling would be required for the evaluation of these structures.

Excellent agreement in the visual evaluation of medial temporal lobe atrophy between the original MPRAGE and generated images (quadratic weighted Cohen kappa values > 0.8 for all raters) suggested that visual assessment may be useful even when only limited sequences are taken, such as in acute stroke examination. On the other hand, further evaluation is needed regarding the detection of cerebrovascular disease between the original MPRAGE and generated images. Cerebrovascular disorders such as acute stroke are usually evaluated with T2-weighted, FLAIR, and diffusion-weighted imaging, and T1-weighted images alone as in this case may not be appropriate as a reading setting.

As shown in Fig. 4, “normal-looking” translations are observed in the generated images. This limits the utility of the present model, and evaluation for brain lesions such as cerebrovascular disorders requires careful evaluation. This problem can occur in other image-to-image transformations using DL [11, 15]. Revisiting this issue with more datasets and/or future advances in DL technology is desirable.

Several limitations to this retrospective study need to be acknowledged. First, although three different models of MR scanner were used, this was a single-vendor, single-center study. A multi-vendor, multi-center evaluation would be desirable to confirm the utility of the model, but was difficult to perform in this study because AAH is the 3D MR localizer used specifically for Siemens MR scanners and the availability of 3D localizer images from other vendors is limited. We explored open databases containing 3D T1-weighted images [27], but none contained AAH as far as we know. Localizer images differ for each vendor, so creating a model specific to each vendor would be desirable. Second, we used only dementia and epilepsy cases. DL relies heavily on training data. Whether similar results can be obtained with healthy subjects or other diseases, such as brain tumors, needs to be examined. Preparing more varied training data may be necessary. Third, we determined the best model based on visual assessments by two radiologists because one purpose of this study was to investigate clinical usefulness. Because some models had very similar image quality, a different model may be selected by different raters. We consider it unlikely that such small differences would result in significant differences in the images generated from the test data and that the results of visual evaluation would differ greatly. Next, the field of DL is advancing rapidly, and various networks have been proposed. We used the pix2pix method in this study, but other networks may be more suitable for this purpose, and there may be points for improvement, such as using a 3D convolutional network. Finally, the visual evaluation assessed cerebrovascular lesions, which are usually evaluated with other sequences such as diffusion-weighted, T2-weighted, and FLAIR imaging. In this study, even with MPRAGE, variations in the detection of cerebrovascular lesions existed between raters.

In conclusion, our DL model for generating MPRAGE-like images from head MR localizer images had the advantage of generating images in post-processing and allowing visual evaluation of medial temporal lobe atrophy. We hope advances in DL methods will reduce erroneous conversions in the future. In the meantime, our approach may become helpful in clinical practice for radiologists familiar with the characteristics of both real and generated images.

## Supplementary Information

Below is the link to the electronic supplementary material.

Supplementary file1 (DOCX 6035 KB)
