# Supplementary material for: Generation of high-resolution MPRAGE-like images from 3D head MRI localizer (AutoAlign Head) images using a deep learning-based model
Source: Jpn J Radiol. 2025 Jan 11;43(5):761–9. doi: 10.1007/s11604-024-01728-8 (PMC12053187; doi:10.1007/s11604-024-01728-8)
Supplement: Supplementary file 1 — Supplementary file1 (DOCX 6035 KB) [file 11604_2024_1728_MOESM1_ESM.docx]

**Supplementary Table 1.** PSNR (mean ± SD) between MPRAGE and generated images of the validation dataset

| $\lambda_{L1}$ | $\lambda_{P}$ | Epoch 10 | Epoch 20 | Epoch 30 | Epoch 40 |
| --- | --- | --- | --- | --- | --- |
| 100 | 0 | 33.3 ± 4.47 | 33.3 ± 4.40 | 32.8 ± 4.54 | 32.8 ± 4.38 |
| 100 | 100 | 33.8 ± 4.48 | 34.2 ± 4.61 | 34.4 ± 4.77 | 33.9 ± 4.51 |
| 100 | 699 | 34.1 ± 4.46 | 34.1 ± 4.64 | 34.5 ± 4.70 | 34.9 ± 5.01 |
| 100 | 1000 | 34.9 ± 4.92 | 34.5 ± 4.70 | 34.4 ± 4.79 | 34.8 ± 4.86 |
| 100 | 6999 | 34.5 ± 4.50 | 34.9 ± 4.44 | 35.1 ± 4.75 | 34.9 ± 4.59 |
| 100 | 10,000 | 35.1 ± 4.83 | 34.9 ± 4.68 | 35.2 ± 4.71 | 35.4 ± 4.85 |
| 699 | 0 | 35.0 ± 4.74 | 34.8 ± 4.47 | 35.0 ± 4.74 | 35.0 ± 4.77 |
| 699 | 100 | 35.0 ± 4.73 | 34.8 ± 4.62 | 35.0 ± 4.51 | 35.1 ± 4.63 |
| 699 | 699 | 34.8 ± 4.77 | 35.4 ± 5.02 | **35.1 ± 5.04** | 35.4 ± 5.02 |
| 699 | 1000 | 34.6 ± 4.79 | 35.1 ± 4.88 | 34.5 ± 4.48 | 35.3 ± 5.03 |
| 699 | 6999 | 35.0 ± 4.74 | 35.2 ± 4.60 | 35.4 ± 4.61 | 35.4 ± 4.88 |
| 699 | 10,000 | 35.4 ± 4.80 | 35.4 ± 4.90 | 35.6 ± 4.95 | 35.7 ± 5.14 |
| 1000 | 0 | 35.3 ± 4.97 | 35.6 ± 4.93 | 35.1 ± 4.76 | 35.3 ± 4.92 |
| 1000 | 100 | 35.0 ± 4.60 | 34.9 ± 4.77 | 35.1 ± 4.64 | 34.9 ± 4.57 |
| 1000 | 699 | 35.3 ± 4.96 | 34.6 ± 4.64 | 35.6 ± 5.09 | 35.7 ± 5.10 |
| 1000 | 1000 | 35.3 ± 5.12 | 34.8 ± 4.95 | 35.5 ± 4.99 | 35.3 ± 4.80 |
| 1000 | 6999 | 34.5 ± 4.50 | 34.9 ± 4.44 | 35.1 ± 4.75 | 34.9 ± 4.59 |
| 1000 | 10,000 | 35.2 ± 4.91 | 35.4 ± 4.95 | 35.6 ± 4.92 | 35.6 ± 4.90 |
| 6999 | 0 | 36.6 ± 5.17 | 35.9 ± 4.35 | 36.1 ± 4.51 | 36.3 ± 4.61 |
| 6999 | 100 | 36.3 ± 5.12 | 35.8 ± 4.72 | 36.1 ± 4.57 | 35.9 ± 4.47 |
| 6999 | 699 | 36.1 ± 5.00 | 36.0 ± 4.78 | 35.9 ± 4.71 | 35.9 ± 4.71 |
| 6999 | 1000 | 35.6 ± 4.66 | 35.9 ± 4.88 | 35.6 ± 4.47 | 35.9 ± 4.86 |
| 6999 | 6999 | 36.1 ± 5.04 | 35.9 ± 4.73 | 36.1 ± 4.90 | 36.0 ± 4.65 |
| 6999 | 10,000 | 35.5 ± 4.65 | 35.9 ± 4.87 | 36.0 ± 4.96 | 36.2 ± 5.04 |
| 10,000 | 0 | 36.8 ± 5.32 | 37.0 ± 5.28 | 37.1 ± 5.35 | 36.9 ± 5.24 |
| 10,000 | 100 | 36.7 ± 5.26 | 36.7 ± 4.98 | 36.5 ± 4.99 | 36.5 ± 4.88 |
| 10,000 | 699 | 36.0 ± 4.88 | 36.1 ± 4.74 | 36.1 ± 4.68 | 36.0 ± 4.60 |
| 10,000 | 1000 | 36.0 ± 5.00 | 36.2 ± 4.77 | 36.1 ± 4.89 | 36.2 ± 4.65 |
| 10,000 | 6999 | 35.7 ± 4.63 | 36.3 ± 5.06 | 36.2 ± 4.97 | 36.2 ± 4.96 |
| 10,000 | 10,000 | 35.5 ± 4.61 | 36.1 ± 5.01 | 36.1 ± 5.02 | 36.0 ± 4.91 |

PSNR, peak signal-to-noise ratio (higher values are better); SD, standard deviation. $\lambda_{L1}$ and $\lambda_{P}$ are hyperparameters representing evaluation function weights for L1 loss and VGG perceptual loss, respectively. This study adopted values of: $\lambda_{L1}$, 699; $\lambda_{P}$, 699; and epochs, 30.

**Supplementary Table 2.** SSIM (mean ± SD) between MPRAGE and generated images of the validation dataset

| $\lambda_{L1}$ | $\lambda_{P}$ | Epoch 10 | Epoch 20 | Epoch 30 | Epoch 40 |
| --- | --- | --- | --- | --- | --- |
| 100 | 0 | 0.829 ± 0.067 | 0.833 ± 0.068 | 0.833 ± 0.068 | 0.830 ± 0.068 |
| 100 | 100 | 0.851 ± 0.064 | 0.855 ± 0.064 | 0.852 ± 0.065 | 0.852 ± 0.066 |
| 100 | 699 | 0.863 ± 0.060 | 0.864 ± 0.061 | 0.866 ± 0.060 | 0.868 ± 0.061 |
| 100 | 1000 | 0.862 ± 0.060 | 0.864 ± 0.059 | 0.868 ± 0.059 | 0.868 ± 0.060 |
| 100 | 6999 | 0.869 ± 0.057 | 0.873 ± 0.057 | 0.875 ± 0.057 | 0.877 ± 0.056 |
| 100 | 10,000 | 0.872 ± 0.057 | 0.874 ± 0.056 | 0.877 ± 0.056 | 0.878 ± 0.056 |
| 699 | 0 | 0.859 ± 0.063 | 0.861 ± 0.062 | 0.863 ± 0.061 | 0.862 ± 0.062 |
| 699 | 100 | 0.864 ± 0.061 | 0.864 ± 0.061 | 0.868 ± 0.060 | 0.867 ± 0.061 |
| 699 | 699 | 0.867 ± 0.060 | 0.871 ± 0.059 | **0.873 ± 0.059** | 0.873 ± 0.059 |
| 699 | 1000 | 0.867 ± 0.060 | 0.870 ± 0.058 | 0.871 ± 0.058 | 0.872 ± 0.059 |
| 699 | 6999 | 0.873 ± 0.056 | 0.876 ± 0.055 | 0.879 ± 0.055 | 0.879 ± 0.056 |
| 699 | 10,000 | 0.874 ± 0.057 | 0.877 ± 0.056 | 0.878 ± 0.056 | 0.879 ± 0.056 |
| 1000 | 0 | 0.861 ± 0.061 | 0.866 ± 0.060 | 0.865 ± 0.062 | 0.864 ± 0.062 |
| 1000 | 100 | 0.865 ± 0.060 | 0.865 ± 0.060 | 0.867 ± 0.061 | 0.866 ± 0.061 |
| 1000 | 699 | 0.868 ± 0.059 | 0.871 ± 0.059 | 0.873 ± 0.059 | 0.874 ± 0.059 |
| 1000 | 1000 | 0.869 ± 0.059 | 0.871 ± 0.058 | 0.876 ± 0.058 | 0.876 ± 0.057 |
| 1000 | 6999 | 0.869 ± 0.057 | 0.873 ± 0.057 | 0.875 ± 0.057 | 0.877 ± 0.056 |
| 1000 | 10,000 | 0.872 ± 0.056 | 0.876 ± 0.056 | 0.878 ± 0.056 | 0.880 ± 0.056 |
| 6999 | 0 | 0.875 ± 0.053 | 0.880 ± 0.050 | 0.882 ± 0.052 | 0.884 ± 0.051 |
| 6999 | 100 | 0.876 ± 0.053 | 0.877 ± 0.051 | 0.883 ± 0.051 | 0.882 ± 0.052 |
| 6999 | 699 | 0.877 ± 0.054 | 0.882 ± 0.052 | 0.882 ± 0.051 | 0.882 ± 0.051 |
| 6999 | 1000 | 0.876 ± 0.055 | 0.881 ± 0.053 | 0.881 ± 0.051 | 0.881 ± 0.054 |
| 6999 | 6999 | 0.880 ± 0.055 | 0.880 ± 0.054 | 0.883 ± 0.054 | 0.883 ± 0.054 |
| 6999 | 10,000 | 0.876 ± 0.056 | 0.880 ± 0.055 | 0.881 ± 0.055 | 0.883 ± 0.054 |
| 10,000 | 0 | 0.882 ± 0.052 | 0.885 ± 0.053 | 0.888 ± 0.053 | 0.886 ± 0.053 |
| 10,000 | 100 | 0.877 ± 0.053 | 0.886 ± 0.052 | 0.885 ± 0.052 | 0.887 ± 0.052 |
| 10,000 | 699 | 0.878 ± 0.053 | 0.885 ± 0.051 | 0.886 ± 0.051 | 0.886 ± 0.052 |
| 10,000 | 1000 | 0.880 ± 0.053 | 0.886 ± 0.052 | 0.884 ± 0.050 | 0.887 ± 0.051 |
| 10,000 | 6999 | 0.880 ± 0.054 | 0.883 ± 0.054 | 0.885 ± 0.054 | 0.886 ± 0.053 |
| 10,000 | 10,000 | 0.876 ± 0.054 | 0.882 ± 0.055 | 0.883 ± 0.053 | 0.884 ± 0.053 |

SSIM, structural similarity index measure (higher values are better); SD, standard deviation. $\lambda_{L1}$ and $\lambda_{P}$ are hyperparameters representing evaluation function weights for L1 loss and VGG perceptual loss, respectively. This study adopted values of: $\lambda_{L1}$, 699; $\lambda_{P}$, 699; and epochs, 30.

**Supplementary Table 3.** LPIPS (mean ± SD) between MPRAGE and generated images of the validation dataset

| $\lambda_{L1}$ | $\lambda_{P}$ | Epoch 10 | Epoch 20 | Epoch 30 | Epoch 40 |
| --- | --- | --- | --- | --- | --- |
| 100 | 0 | 0.060 ± 0.020 | 0.058 ± 0.022 | 0.059 ± 0.022 | 0.059 ± 0.022 |
| 100 | 100 | 0.052 ± 0.019 | 0.050 ± 0.017 | 0.049 ± 0.017 | 0.050 ± 0.019 |
| 100 | 699 | 0.048 ± 0.017 | 0.047 ± 0.017 | 0.045 ± 0.016 | 0.045 ± 0.017 |
| 100 | 1000 | 0.046 ± 0.017 | 0.045 ± 0.016 | 0.045 ± 0.017 | 0.045 ± 0.016 |
| 100 | 6999 | 0.047 ± 0.016 | 0.046 ± 0.016 | 0.045 ± 0.016 | 0.044 ± 0.016 |
| 100 | 10,000 | 0.058 ± 0.023 | 0.058 ± 0.023 | 0.057 ± 0.022 | 0.057 ± 0.022 |
| 699 | 0 | 0.050 ± 0.019 | 0.049 ± 0.018 | 0.048 ± 0.018 | 0.048 ± 0.018 |
| 699 | 100 | 0.046 ± 0.017 | 0.045 ± 0.016 | 0.045 ± 0.016 | 0.044 ± 0.016 |
| 699 | 699 | 0.045 ± 0.017 | 0.044 ± 0.017 | **0.044 ± 0.017** | 0.044 ± 0.016 |
| 699 | 1000 | 0.046 ± 0.017 | 0.044 ± 0.016 | 0.045 ± 0.016 | 0.045 ± 0.017 |
| 699 | 6999 | 0.054 ± 0.019 | 0.050 ± 0.018 | 0.051 ± 0.018 | 0.051 ± 0.018 |
| 699 | 10,000 | 0.056 ± 0.023 | 0.055 ± 0.022 | 0.056 ± 0.022 | 0.055 ± 0.021 |
| 1000 | 0 | 0.048 ± 0.019 | 0.049 ± 0.019 | 0.047 ± 0.018 | 0.047 ± 0.018 |
| 1000 | 100 | 0.045 ± 0.017 | 0.045 ± 0.017 | 0.045 ± 0.017 | 0.045 ± 0.017 |
| 1000 | 699 | 0.045 ± 0.016 | 0.044 ± 0.016 | 0.043 ± 0.016 | 0.043 ± 0.016 |
| 1000 | 1000 | 0.045 ± 0.016 | 0.044 ± 0.016 | 0.044 ± 0.017 | 0.043 ± 0.016 |
| 1000 | 6999 | 0.047 ± 0.016 | 0.046 ± 0.016 | 0.045 ± 0.016 | 0.044 ± 0.016 |
| 1000 | 10,000 | 0.058 ± 0.020 | 0.055 ± 0.020 | 0.056 ± 0.020 | 0.055 ± 0.020 |
| 6999 | 0 | 0.055 ± 0.028 | 0.065 ± 0.035 | 0.065 ± 0.036 | 0.068 ± 0.037 |
| 6999 | 100 | 0.050 ± 0.021 | 0.054 ± 0.029 | 0.065 ± 0.034 | 0.061 ± 0.032 |
| 6999 | 699 | 0.044 ± 0.018 | 0.047 ± 0.021 | 0.049 ± 0.024 | 0.050 ± 0.024 |
| 6999 | 1000 | 0.045 ± 0.017 | 0.045 ± 0.018 | 0.045 ± 0.020 | 0.047 ± 0.021 |
| 6999 | 6999 | 0.057 ± 0.022 | 0.056 ± 0.021 | 0.055 ± 0.022 | 0.055 ± 0.022 |
| 6999 | 10,000 | 0.056 ± 0.020 | 0.055 ± 0.019 | 0.055 ± 0.019 | 0.054 ± 0.019 |
| 10,000 | 0 | 0.066 ± 0.033 | 0.080 ± 0.035 | 0.083 ± 0.033 | 0.084 ± 0.033 |
| 10,000 | 100 | 0.053 ± 0.026 | 0.070 ± 0.034 | 0.067 ± 0.029 | 0.075 ± 0.031 |
| 10,000 | 699 | 0.048 ± 0.019 | 0.057 ± 0.027 | 0.058 ± 0.028 | 0.058 ± 0.028 |
| 10,000 | 1000 | 0.048 ± 0.019 | 0.054 ± 0.026 | 0.055 ± 0.027 | 0.057 ± 0.028 |
| 10,000 | 6999 | 0.056 ± 0.022 | 0.055 ± 0.022 | 0.054 ± 0.022 | 0.053 ± 0.021 |
| 10,000 | 10,000 | 0.056 ± 0.020 | 0.055 ± 0.020 | 0.055 ± 0.020 | 0.055 ± 0.020 |

LPIPS, Learned Perceptual Image Patch Similarity (lower values are better); SD, standard deviation. $\lambda_{L1}$ and $\lambda_{P}$ are hyperparameters representing evaluation function weights for L1 loss and VGG perceptual loss, respectively. This study adopted values of: $\lambda_{L1}$, 699; $\lambda_{P}$, 699; and epochs, 30.

**Supplementary Text**

Deep learning procedures

Since pix2pix requires the source and target images to be aligned, the AAH image was first registered to the MPRAGE image using SPM 12 software (https://www.fil.ion.ucl.ac.uk/spm/software/spm12/). AAH and MPRAGE were resliced to transverse images. Only the image containing brain parenchyma was selected as the training target. One radiologist (HT, 10 years of experience in radiology) determined whether brain parenchyma was included or not. Random crop, flip, and image rotation steps were used for data augmentation.

The original pix2pix evaluation function ($G^{*}$) is the following using a combination of GAN ($\mathcal{L}_{cGAN}\left( G, D \right)$) and L1 loss ($\mathcal{L}_{L1}\left( G \right)$) [1].

$$G^{*}=\arg\min_{G} \max_{D} \mathcal{L}_{cGAN}\left( G, D \right) + \lambda_{L1}\mathcal{L}_{L1}\left( G \right)$$

Note that $\arg{min}_{G}{max}_{D}$ means where Generator (G) tries to minimize this objective against Discriminator (D) that tries to maximize the objective.

This study also used VGG perceptual loss ($\mathcal{L}_{p}(G)$) using the pre-trained VGG16 network:

$$G^{*}=\arg\min_{G} \max_{D} \mathcal{L}_{cGAN}\left( G, D \right) + \lambda_{L1}\mathcal{L}_{L1}\left( G \right)+ \lambda_{p}\mathcal{L}_{p}(G)$$

$\lambda_{L1}$ and $\lambda_{p}$ are arbitrary coefficients that determine the weight of loss and can have a substantial impact on image quality. We therefore performed training by varying each coefficient from 0 to 10,000. To improve the training procedure, we introduced one-side label smoothing for GAN; the true label was set to 0.9 from 1 [2].

The learning process was monitored with Weights & Biases (https://www.wandb.ai/) for losses and image evaluation metrics. As a measure of image similarity in the learning process, we mainly used structural similarity index measure (SSIM, higher values are better) and Learned Perceptual Image Patch Similarity (LPIPS, lower values are better). In GAN training, the balance between loss of function like L1 loss, which compares the real and generated images, and the feedback from the discriminator is a key factor. We therefore also monitored L1 loss, VGG perceptual loss, and discriminator loss (Discriminator_real, Discriminator_fake). Discriminator_real indicates whether the discriminator determined that the real MPRAGE was real, and Discriminator_fake indicates whether the discriminator determined that the generated image was fake. To prevent over-fitting, training was terminated when little improvement was seen in loss or evaluation metrics. All learning processes were performed on a workstation installed with Ubuntu 22.04; the GPU was NVIDIA RTX 3080 (10 GB).

**References (Supplementary Text)**

1. Isola PaZ, Jun-Yan and Zhou, Tinghui and Efros, Alexei A. Image-to-Image Translation with Conditional Adversarial Networks. CVPR. 2017.

2. Salimans T, Goodfellow I, Zaremba W, Cheung V, Radford A, Chen X. Improved Techniques for Training GANs. arXiv pre-print server. 2016.

**Supplementary Figure 1**


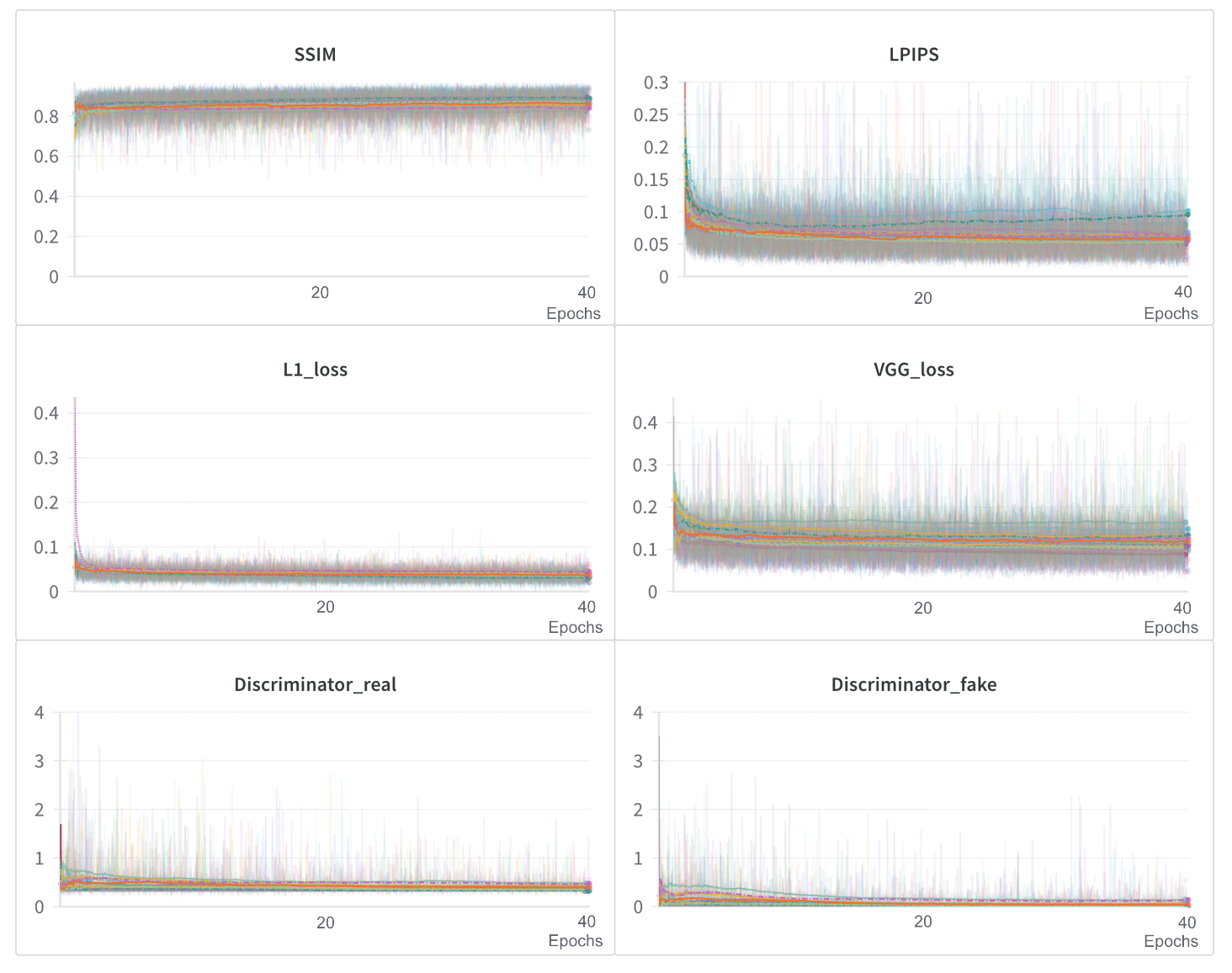


The learning curves of thirty models for validation are shown. Processes were monitored on Weights & Biases (https://wandb.ai/), an AI developer platform. The training was terminated at 40 epochs due to a lack of improvement in the indicator. SSIM, structural similarity index measure (higher values are better); LPIPS, Learned Perceptual Image Patch Similarity (lower values are better). VGG_loss indicates the perceptual loss function using the VGG 16 network; Discriminator_real indicates whether the discriminator determined that the real MPRAGE was real; and Discriminator_fake indicates whether the discriminator determined that the generated image was fake. Note that we used one-side label smoothing, which prevents Discriminator_real from being closer to zero than a specific value. The best model was selected as described in the Model Selection of Materials and Methods.

**Supplementary Figure 2**


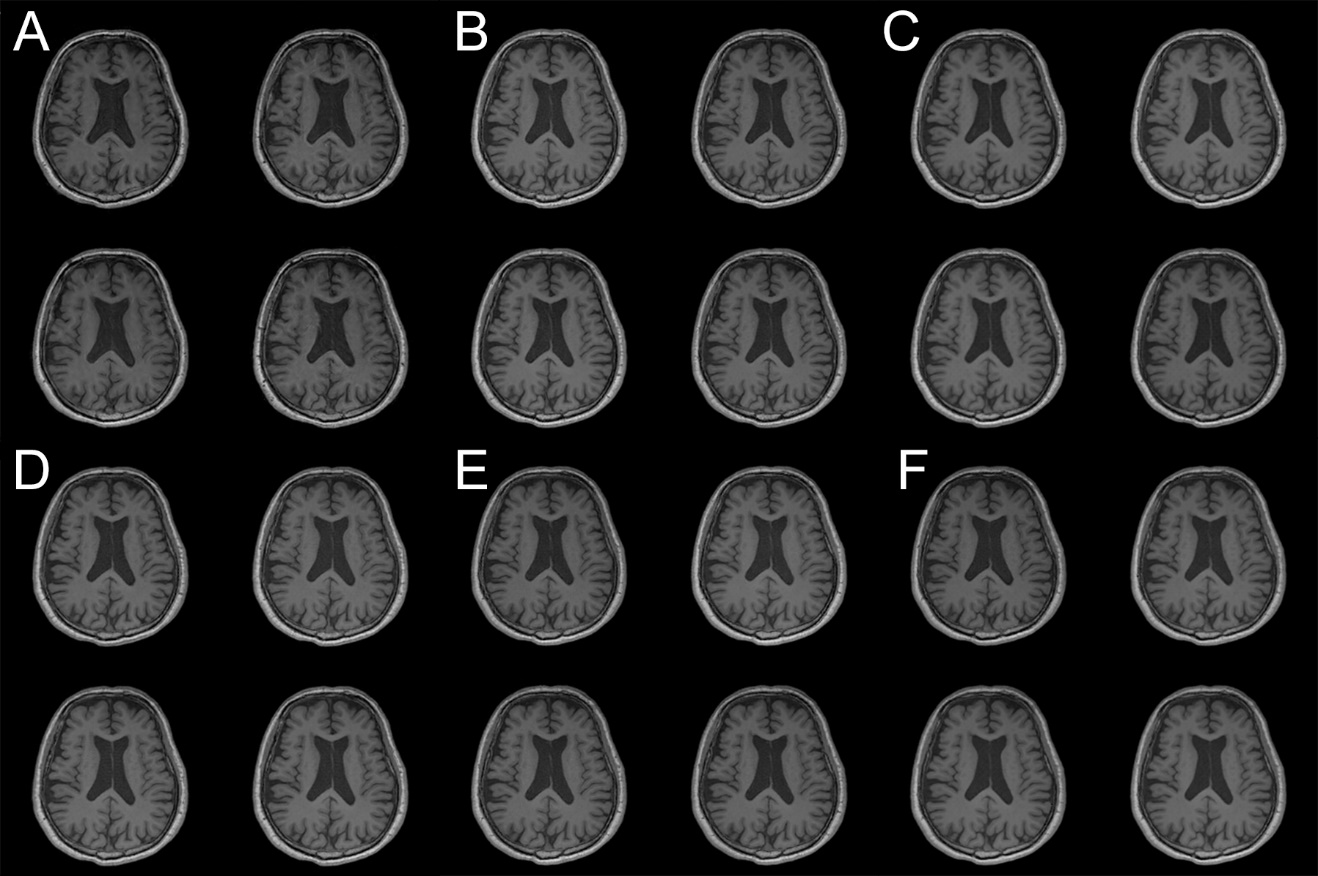


Sample images that are used to select an optimal model for validation datasets. (L_L1, L_P) are (100, 0) [A]; (699, 699) [B] epoch 30 is the selected model; (699, 10,000) [C]; (1000, 1000) [D]; (6999, 1000) [E]; and (10,000, 699) [F]. Note that each condition contains epoch 10 (upper left), 20 (upper right), 30 (lower left), and 40 (lower right). Sample images that are used to select an optimal model for validation datasets. (L_L1, L_P) are (100, 0) [A]; (699, 699) [B] epoch 30 is the selected model; (699, 10,000) [C]; (1000, 1000) [D]; (6999, 1000) [E]; and (10,000, 699) [F]. Note that each condition contains epoch 10 (upper left), 20 (upper right), 30 (lower left), and 40 (lower right). $\lambda_{L1}$ represents L1 loss weights, and $\lambda_{P}$ represents VGG perceptual loss weights for the evaluation function.

**Supplementary Figure 3**


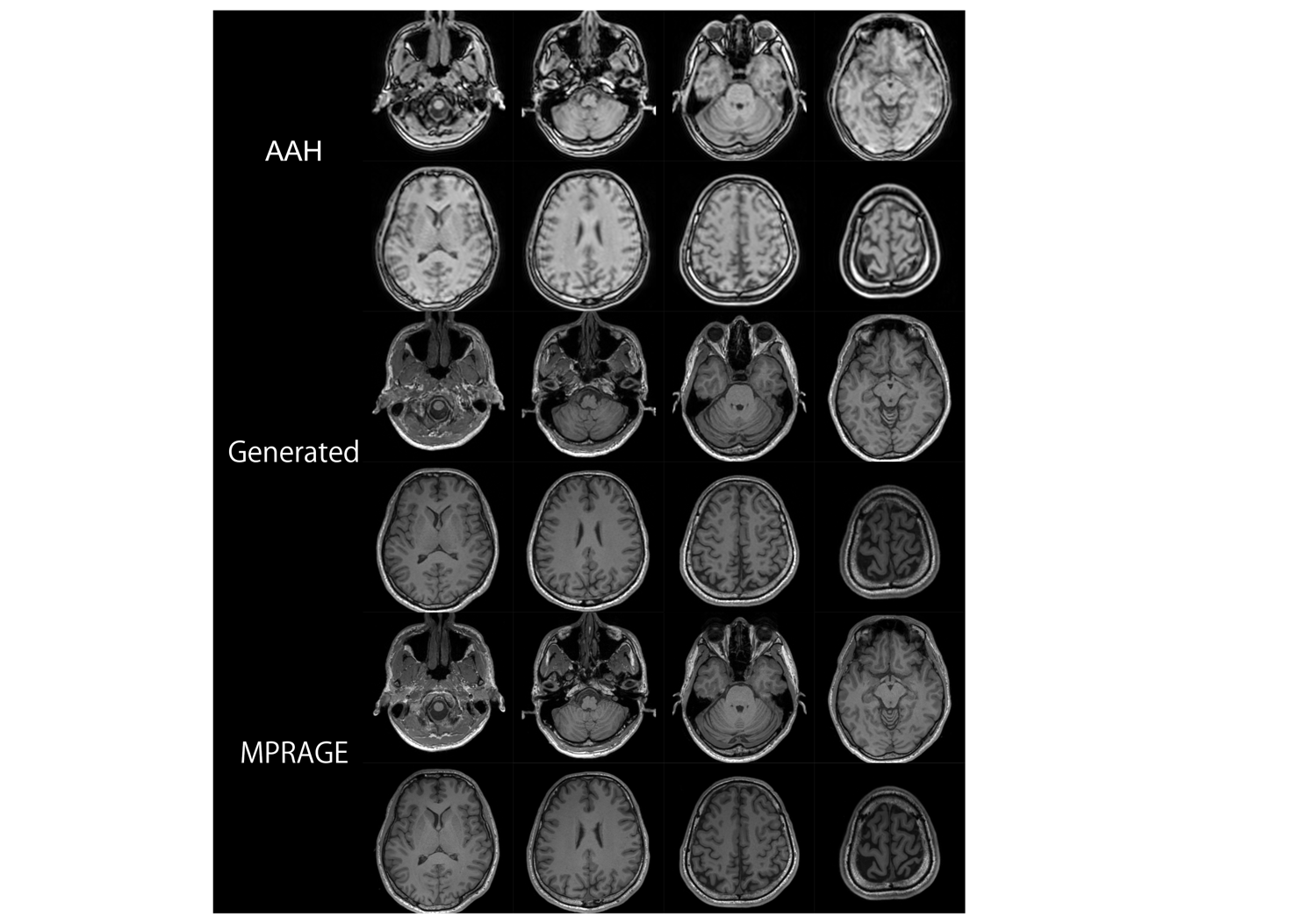


Representative whole-brain images from test datasets.
